# Editorial: Narrating exile

**DOI:** 10.3389/fsoc.2024.1347417

**Published:** 2024-01-30

**Authors:** Siobhan Holohan, Ala Sirriyeh

**Affiliations:** ^1^Department of Sociology, Keele University, Staffordshire, United Kingdom; ^2^Department of Sociology, Lancaster University, Lancaster, United Kingdom

**Keywords:** exile, migration, belonging, exilic consciousness, liminality

The concept of migration as an exilic condition was perhaps most famously articulated in Arendt's (1943) influential essay “We Refugees,” which described a situation where Jewish refugees were stripped of identity and reconstructed as other wherever they went. With no political sovereignty, the Jewish diaspora were forced to surrender their identity to the demands of whichever political community granted sanctuary. For Arendt, rejection of self was the price to be paid for being granted human rights. In this regard, the refugee is always written in terms of what Lacan (2007) calls lack, and the exile comes to embody the abject position forced upon them. Against this position of lack, in her later work Arendt (1958) argued that the identity of the exile exists as an essential form: natality or personal sovereignty. Here, she says that natality overwrites the national legal-political complex of state law and the nationalistic discourses that seek to undermine the identity of the exile to form a new kind of belonging based on based on a historical-culturally constructed notion of collective belonging (Holohan, 2019).

Said's (2000, 2012) reflections on the Palestinian condition rearticulate the pain of exile experienced by refugees and migrants as a force with the ability to motivate and liberate the subject from their position of lack (Bernard, 2014). Here, he extends the idea of exilic consciousness as a condition where the displaced experience psychic disconnection from place and selfhood. For Said, exilic consciousness can help to explain the contradictions contained with the migration experience often articulated in the articles in this Research Topic. Yet, between these contradictions is revealed a tacit understanding of the underlying irrationality of the exilic situation; one where belonging is contingent on the gifting of the right to live well. But for Said exilic consciousness is also about being able to imagine otherwise. Here, he describes the exile as a liminal intellectual, whose experience encourages the consideration of a possible future despite the reality of the present. This signifies hope, that elusive element that motivates us to endure even when we believe our capabilities are stretched to the limit.

This sense of hope/hopelessness is described in Sagbakken et al.'s analysis of the health outcomes of asylum seekers and undocumented migrants in Norway. They describe the experience of the asylum system as “waiting for nothing,” a condition that contributes to poor physical and mental health, where the anomic experience of statelessness induces loneliness, self-harm, and suicidal thoughts. Yet, despite this, participants remained hopeful of the outcome “next time.” This sense of hope might be described as a “coping-strategy” following El Masri's poignant commentary on the challenging situation of the long-standing Palestinian refugee community in Lebanon. Here, refugees exist in a condition of “permanently-temporary” statelessness and face high rates of poverty and State/Third-Sector intervention. In the face of this refugees attempt to both establish the camp spaces as home and to assert a form of liminal autonomy. By asserting control over the physical infrastructure and informal economic systems, and partaking in community bonding practices through storytelling, they establish a tolerable existence within these “waiting zones.”

While participants in Sagbakken et al.'s and El Masri's study continue to hope for a better outcome, the authors question whether the structural violence experienced by non-citizens is intentional on the part of the State. Contini and Carrera emphasize the role of power-holders in their discussion of migration in terms of the urban landscape within which encounters between settled and migrant communities take place. Set against an interrogation of the complex histories of migration, the authors reimagine the role of the policymaker in facilitating positive communication between communities through the development of “third spaces [as an] urban experience of crossings, of going beyond borders, which can generate inclusive practices of meeting and cultural contamination.” Contini and Carrera suggest that the development of third spaces opens up the possibility for the form of hybridization conceived by Bhabha (1994), which is further articulated in the article by Holle et al.. Despite being excluded in terms of their migration status, queer refugees in the Netherlands are able to mobilize their LGBTQ+ status within a social system that forwards a homonormative discourse of inclusion. Here, the authors argue that queer refugees disrupt nationalistic anti-migrant sentiment and assert a form of liminal agency through resistance through exilic art practices.

We again find the negotiation of identity between migrant/exile and nation/State in the final two articles of the Research Topic. Cakmak's examination of Turkish-speaking communities in North London (UK) unpacks the myth of return common to migration discourses. While the myth of return represents the desire to return to an imagined homeland, the author notes a change influenced by migrants' settled status. Instead of a permanent return, participants in his fieldwork articulate contentment with occasional visits and frequent interactions afforded by contemporary communications networks. Here, identity and belonging are contained within a series of negotiations that are also expressed in Elsayed's exploration of the integration experiences UK-born Muslims. Set against a national discourse of self-segregation, Elsayed contends that the layers of meaning produced by settled migrants offer insight into the often contradictory nature of belonging and non-belonging. Here, she suggests that Said's conceptualization of exilic consciousness helps to understand the contradictions inherent in the migrant experience. Within this space of exilic consciousness, settled second and third generation migrants reveal a tacit comprehension of the underlying injustice of their situation while contemplating a potential, more optimistic future for themselves.

The views of Arendt and Said are conveyed across the articles in this Research Topic, which, in different ways, reveal how the migrant is both subjected to meaning and how exilic identity is constructed against a complex interplay of context-specific factors. Authors explore the conditions and narratives of exile experienced by settled migrants, refugees, asylum seekers, and undocumented migrants, recognizing the impact of public sphere narratives on the everyday experiences of migrant belonging, as well as the potential for resisting dominant construction of otherness and reimagining selfhood through community-building in their borrowed homelands.

## Author contributions

SH: Writing – original draft. AS: Writing – review & editing.
